# Oncological outcomes of neoadjuvant chemotherapy in patients with locally advanced upper tract urothelial carcinoma: a multicenter study

**DOI:** 10.18632/oncotarget.21551

**Published:** 2017-10-06

**Authors:** Yuka Kubota, Shingo Hatakeyama, Toshikazu Tanaka, Naoki Fujita, Hiromichi Iwamura, Jotaro Mikami, Hayato Yamamoto, Yuki Tobisawa, Tohru Yoneyama, Takahiro Yoneyama, Yasuhiro Hashimoto, Takuya Koie, Hiroyuki Ito, Kazuaki Yoshikawa, Atsushi Sasaki, Toshiaki Kawaguchi, Chikara Ohyama

**Affiliations:** ^1^ Department of Urology, Hirosaki University Graduate School of Medicine, Hirosaki, Japan; ^2^ Department of Urology, Tohoku Medical and Pharmaceutical University, Sendai, Japan; ^3^ Department of Advanced Transplant and Regenerative Medicine, Hirosaki University Graduate School of Medicine, Hirosaki, Japan; ^4^ Department of Urology, Aomori Rosai Hospital, Hachinohe, Japan; ^5^ Department of Urology, Mutsu General Hospital, Mutsu, Japan; ^6^ Department of Urology, Tsugaru General Hospital, Goshogawara, Japan; ^7^ Department of Urology, Aomori Prefectural Central Hospital, Aomori, Japan

**Keywords:** carboplatin, chemotherapy, cisplatin, neoadjuvant, upper tract urothelial carcinoma

## Abstract

**Objective:**

The clinical impact of neoadjuvant chemotherapy (NAC) on oncological outcomes in patients with locally advanced upper tract urothelial carcinoma (UTUC) remains unclear. We investigated the oncological outcomes of platinum-based NAC for locally advanced UTUC.

**Results:**

Of 234 patients, 101 received NAC (NAC group) and 133 did not (Control [Ctrl] group). The regimens in the NAC group included gemcitabine and carboplatin (75%), and gemcitabine and cisplatin (21%). Pathological downstagings of the primary tumor and lymphovascular invasion were significantly improved in the NAC than in the Ctrl groups. NAC for locally advanced UTUC significantly prolonged recurrence-free and cancer-specific survival. Multivariate Cox regression analysis using an inverse probability of treatment weighted (IPTW) method showed that NAC was selected as an independent predictor for prolonged recurrence-free and cancer-specific survival. However, the influence of NAC on overall survival was not statistically significant.

**Materials and Methods:**

A total of 426 patients who underwent radical nephroureterectomy at five medical centers between January 1995 and April 2017 were examined retrospectively. Of the 426 patients, 234 were treated for a high-risk disease (stages cT3–4 or locally advanced [cN+] disease) with or without NAC. NAC regimens were selected based on eligibility of cisplatin. We retrospectively evaluated post-therapy pathological downstaging, lymphovascular invasion, and prognosis stratified by NAC use. Multivariate Cox regression analysis was performed for independent factors for prognosis.

**Conclusions:**

Platinum-based NAC for locally advanced UTUC potentially improves oncological outcomes. Further prospective studies are needed to clarify the clinical benefit of NAC for locally advanced UTUC.

## INTRODUCTION

Upper tract urothelial carcinoma (UTUC) is uncommon [1], and the prognosis for high-stage UTUC has not improved over the past two decades [2]. Adjuvant chemotherapy has been considered a therapeutic option; however, loss of renal function after radical nephroureterectomy decreases the eligibility for cisplatin-based chemotherapy [3, 4]. This evidence strongly suggests a need for changing the treatment protocol. A multimodal approach including neoadjuvant chemotherapy (NAC) and surgical resection might improve patient outcomes. However, only a few prospective studies are available regarding the benefit of NAC for UTUC [4, 5] and to our knowledge, no definitive recommendation exists because of insufficient evidence, such as data on appropriate regimens [4, 6]. The benefit of NAC for locally advanced UTUC has been debated based on studies of bladder cancer indicating improved survival after cisplatin-based NAC [7]. Several retrospective studies have addressed the prognostic benefit of NAC for UTUC [8–13]. Recently, we reported the oncological benefits of NAC for locally advanced UTUC in a single-center study [14]. However, the limited number of patients prohibited the conclusion of clinical benefit of NAC for locally advanced UTUC.

We compared oncological outcomes (intravesical recurrence-free survival [RFS], visceral RFS, cancer-specific survival [CSS], and overall survival [OS]) between patients with and without NAC for locally advanced UTUC in a multicenter setting.

## RESULTS

### Baseline characteristics

**Figure 1:**
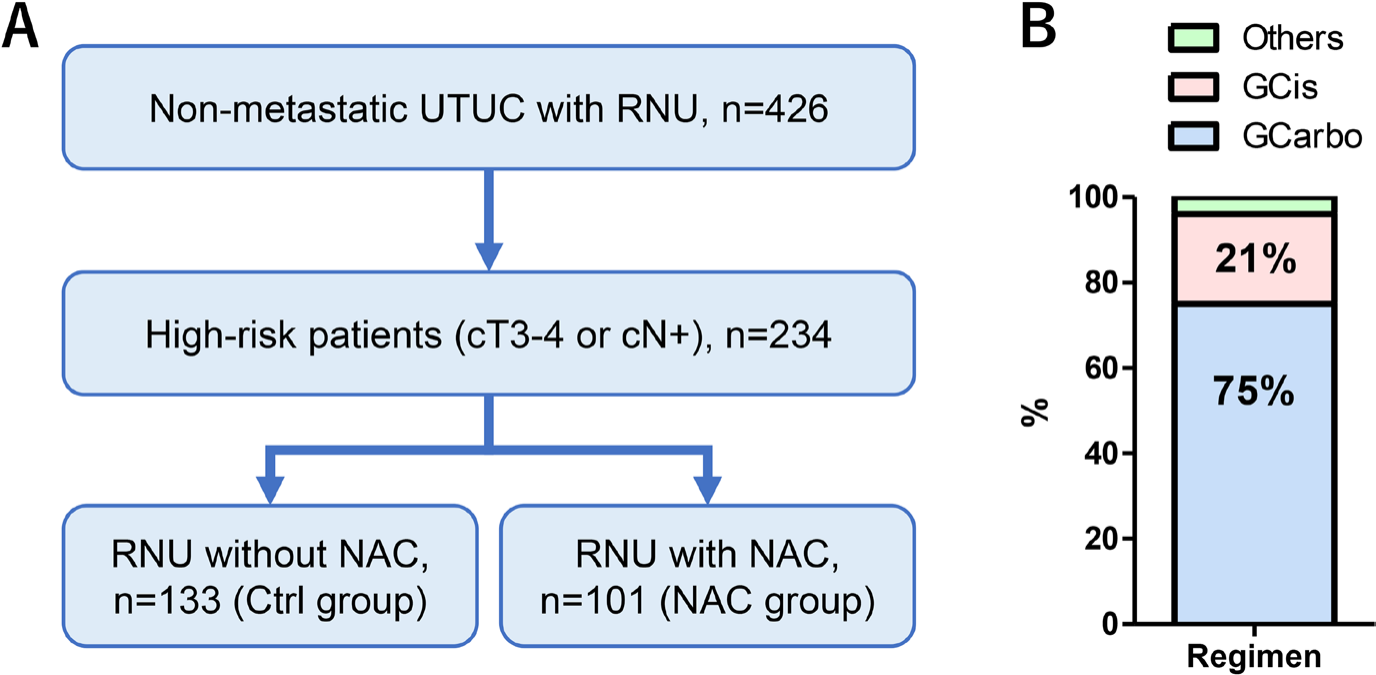
Patient selection and classification Of the 426 patients who underwent radical nephroureterectomy for UTUC, 234 were identified as having high-risk disease (stages cT3-4 or cN+ disease). Of those patients, 101 received 2 to 4 courses of NAC (NAC group) and 133 did not (Ctrl group) (**A**). The regimens in the NAC group were GCarbo in 76 patients (75%) and GCis in 21 (21%) (**B**).

Among 426 patients, the number of the patients with cTis, cT1, cT2, cT3, cT4, and cN+ were 2, 92, 103, 217, 12 and 34, respectively. We identified 234 high-risk patients who received either 2 to 4 courses of NAC (*n* = 101, NAC group) or surgery alone (*n* = 133, Control [Ctrl] group; Figure 1A). There were no significant differences in preoperative patient characteristics between the groups except for tumor location and laparoscopic radical nephroureterectomy (Table 1). The regimens in the NAC group were gemcitabine plus carboplatin (GCarbo) in 76 patients (75%), gemcitabine plus cisplatin (GCis) in 21 (21%), and others (methotrexate, vinblastine, doxorubicin, and cisplatin [MVAC], or a docetaxel-based regimen) in 4 (4%; Figure 1B). The incidence of postoperative complications showed no significant differences between the Ctrl and NAC groups, and no grade 4 or 5 complication was observed (Table 1). Due to the long-term period of the study, the use of laparoscopic surgery was significantly different between the groups (*P* = 0.001).

**Table 1:** Background of patients

	All	Ctrl	NAC	*P* value
*n*	234	133	101	
Age (years)	71 ± 9.2	71 ± 8.9	70 ± 9.5	*0.223*
Gender (Male), *n*=	155 (66%)	85 (64%)	70 (69%)	*0.387*
ECOG-PS > 1, *n* =	5 (2.1%)	4 (3.0%)	1 (1.0%)	*0.393*
Hypertension, *n* =	106 (44%)	65 (49%)	41 (41%)	*0.234*
Diabetes mellitus, *n* =	39 (17%)	19 (14%)	20 (20%)	*0.291*
Cardiovascular disease, *n* =	39 (17%)	23 (17%)	16 (16%)	*0.768*
Smoking, *n* =	102 (44%)	52 (39%)	50 (50%)	*0.112*
eGFR before surgery (mL/min/1.73m^2^)	56 ± 18	56 ± 19	57 ± 15	*0.414*
Hydronephrosis, *n*=	164 (70%)	96 (72%)	68 (67%)	*0.422*
NAC regimen: GCis / GCarbo / others, *n* =			21 /76 /4	
cT 2/3/4, *n* =	5 / 217 / 12	4 / 123 / 6	1 / 94 / 6	*0.566*
cN+, *n* =	34 (15%)	15 (11%)	19 (19%)	*0.105*
Original tumor sites, *n* =				
Renal pelvis / Ureter / Multiple	92 / 125 / 17	61 / 61 / 11	31 / 64 / 6	*0.009*
Laparoscopic surgery, *n* =	48 (21%)	17 (13%)	31 (31%)	*0.001*
Postoperative complications, *n* =				
All	33 (14%)	16 (12%)	17 (17%)	*0.296*
G3	8 (3.4%)	6 (4.5%)	2 (2.0%)	
Pathological outcomes, *n* =				
pT3 or 4	141 (60%)	105 (79%)	36 (36%)	*< 0.00*1
Downstaging	58 (25%)	19 (14%)	39 (39%)	*< 0.001*
Downstaging (cT - pT)	0.6 ± 1.0	0.3 ± 0.8	1.1 ± 1.0	*< 0.001*
pN+	27 (12%)	16 (12%)	11 (11%)	*0.787*
High grade	222 (95%)	127 (96%)	95 (94%)	*0.623*
Concomitant CIS	22 (9.4%)	10 (7.5%)	12 (12%)	*0.257*
Surgical margin positive	14 (6.0%)	9 (6.8%)	5 (5.0%)	*0.562*
Lymphovascular invasion	87 (37%)	61 (46%)	26 (26%)	*0.004*
Median follow-up (Months)	27	30	26	

### Tumor response characteristics

The number of patients with pT3 or 4 disease was significantly higher in the Ctrl (*n* = 105, 79%) than in the NAC (*n* = 36, 36%) groups (*P* < 0.001) (Table 1). The number of patients with downstaging in the Ctrl and NAC groups were 14% and 39% (*P* < 0.001), respectively. The mean number of primary tumors that were pathologically downstaged (cT–pT stage) was significantly higher in the NAC (1.1 ± 1.0) compared to the Ctrl (0.3 ± 0.8) groups (*P* < 0.001). Additionally, the number of patients with lymphovascular invasion (LVI) was significantly lower in the NAC (*n* = 26, 26%) than in the Ctrl (*n* = 61, 46%) groups (*P* = 0.004).

### Oncological outcomes

**Figure 2:**
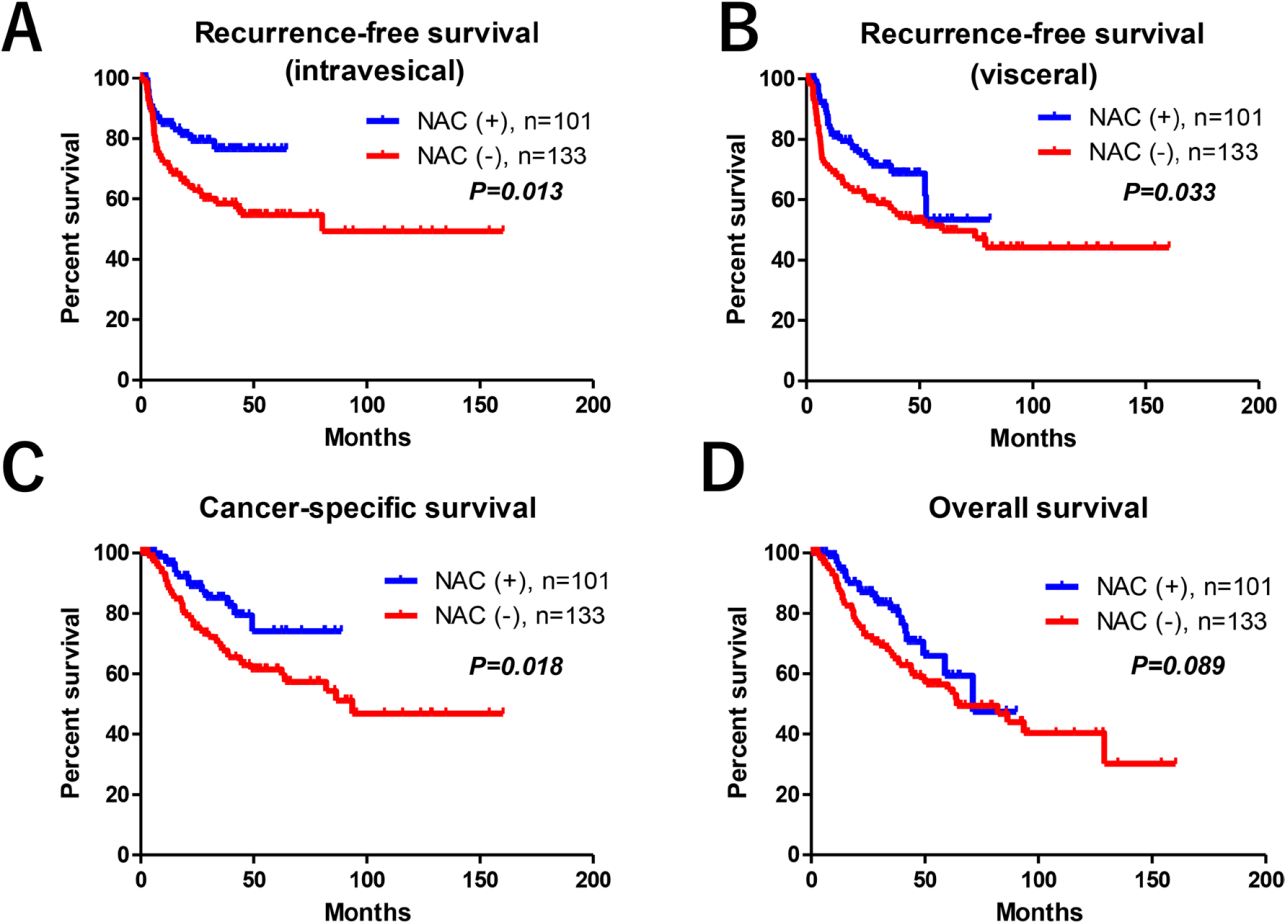
Oncological outcomes There were statistically significant differences in the intravesical RFS (**A**), visceral RFS (**B**), CSS (**C**), but not in OS (**D**) between the two groups.

The median follow-up in the Ctrl and NAC groups was 30 and 26 months, respectively. There were statistically significant differences in the intravesical RFS, visceral RFS, and CSS measures between the Ctrl and NAC groups (Figure 2A–2C). The NAC group had significantly better 5-year intravesical RFS (77% vs. 55%, *P* = 0.013), visceral RFS (53% vs. 50%, *P* = 0.033), and CSS (74% vs. 62%, *P* = 0.018) compared to the Ctrl group. However, no difference was observed in 5-year OS (59% vs. 55%, *P* = 0.089) between the groups (Figure 2D).

### Oncological outcomes between the patients with GCis and GCarbo NAC

**Figure 3:**
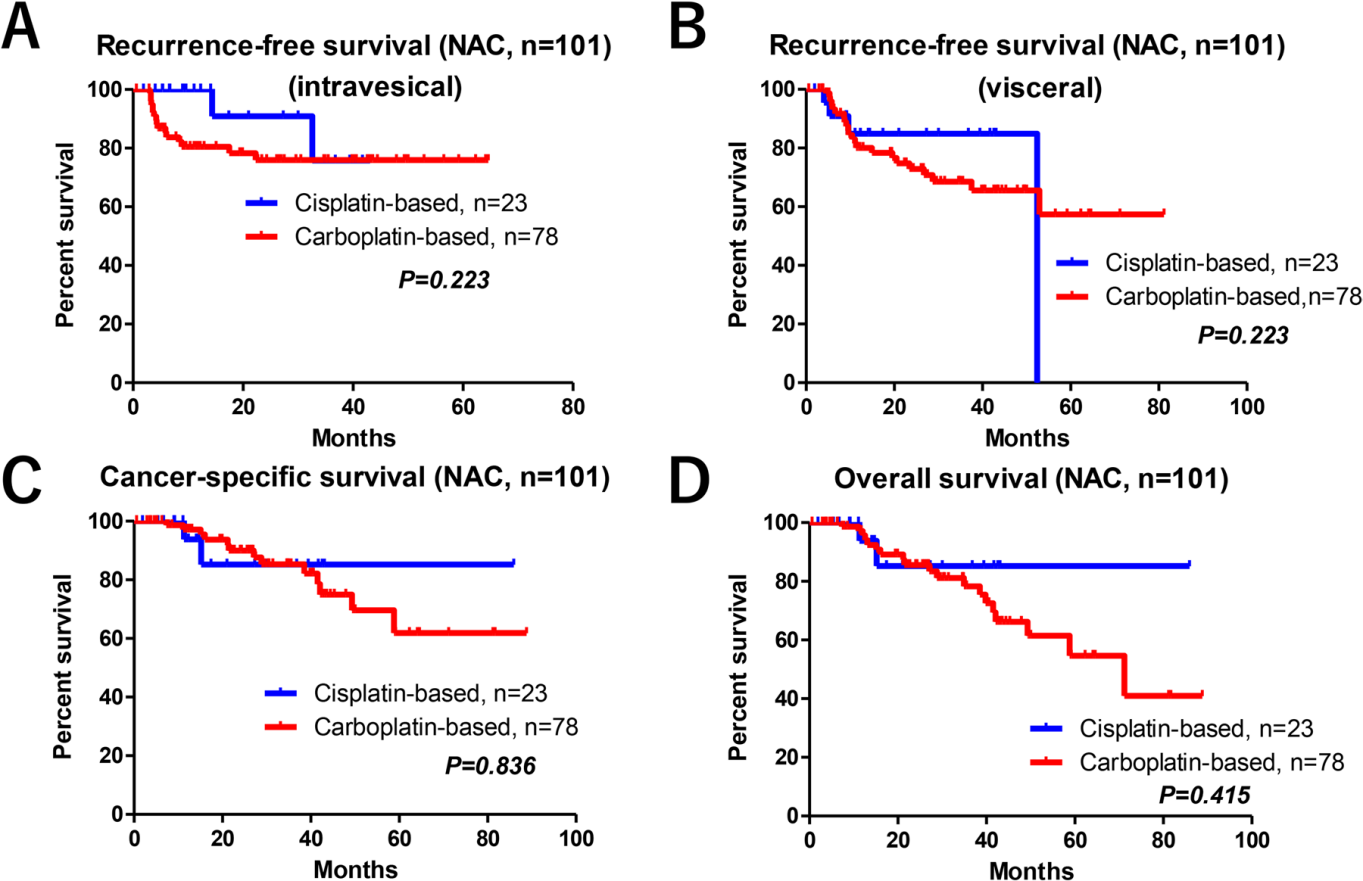
Oncological outcomes between the GCis and GCarbo NAC regimens There were no significant differences in the intravesical RFS (**A**), visceral RFS (**B**), CSS (**C**), and OS (**D**) between patients who received GCis and GCarbo NAC regimens.

Of the 101 patients who received NAC, the median ages for those who were given GCis (66 years; interquartile range [IQR], 61–73) or GCarbo regimens (73 years; IQR, 66–78) were statistically different (*P* = 0.028). The median number of courses of NAC was 2 in both regimens. Due to the patients’ selection for cisplatin-eligibility, the median estimated glomerular filtration rates (eGFRs) were significantly lower in patients who received GCarbo NAC than in those who received GCis therapy (67 vs. 55 mL/min/1.73 m^2^, respectively). However, median radiologic tumor reductions were not statistically different between the GCis (23%; IQR, 12%–45%) and GCarbo (26%; IQR, 2%–41%) therapies, respectively (*P* = 0.543). There were no significant differences between intravesical RFS, visceral RFS, CSS, and OS when comparing the two therapies (Figure 3A–3D).

### Uni- and multivariate analyses for prognosis

In univariate Cox regression analysis, NAC was selected as an independent factor for CSS, although the impacts of NAC on intravesical RFS, visceral RFS, and OS were not significant (Table 2, upper row). Multivariate Cox regression analysis using an inverse probability of treatment weighting (IPTW) model revealed that the impact of NAC on intravesical RFS (*P* = 0.023; hazards ratio [HR], 0.52), visceral RFS (*P* = 0.021; HR, 0.57) and CSS (*P* = 0.016; HR, 0.48) was significant, whereas the impact on OS (*P* = 0.081; HR, 0.62) was not significant (Table 2, lower row).

**Table 2:** Uni- and multivariate Cox regression analyses for prognosis

Univariate	Factor	*P* value	HR	95%CI
Recurrence-free survival (Intravesical relapse: Ta-1)	NAC	*0.233*	0.71	0.41-1.24
Recurrence-free survival (visceral)	NAC	*0.381*	0.81	0.50-1.30
Cancer-specific survival	NAC	*0.021*	0.48	0.26-0.90
Overall survival	NAC	*0.157*	0.69	0.41-1.15
Multivariate (IPTW analysis^*^)	**Factor**	***P*** **value**	**HR**	**95%CI**
Recurrence-free survival (Intravesical relapse: Ta-1)	NAC	*0.023*	0.52	0.30-0.91
Recurrence-free survival (visceral)	NAC	*0.021*	0.57	0.36-0.92
Cancer-specific survival	NAC	*0.016*	0.48	0.26-0.87
Overall survival	NAC	*0.081*	0.62	0.37-1.06

^*^Inverse probability of treatment weighting analysis. Variables included in the IPTW model were age, sex, eGFR, cT, cN. CI: confidence interval.

## DISCUSSION

The essential finding of our study was that NAC for locally advanced UTUC has potential to improve oncological outcomes. Our results suggested that patients with locally advanced UTUC were potential candidates for NAC. Multivariate Cox regression analysis using the IPTW model revealed that the impact of NAC on intravesical RFS, visceral RFS and CSS was significant, but it was not significant on OS (*P* = 0.081). Limited impact of NAC on OS might be due to the elderly population with UTUC and the short-term follow up of the NAC group. These results are consistent with our previous study that reported efficacy and safety of platinum-based NAC for locally advanced UTUC in 51 pair-matched patients [14]. The two arms (without vs. with NAC) were matched using propensity scores to minimize selection bias. The retrospective evaluation of safety, tumor response, post-therapy pathological downstaging, LVI, and prognosis showed feasibility and potential efficacy of NAC for locally advanced UTUC. Pathological downstaging of the primary tumor was significantly higher in patients with NAC than in those without NAC. NAC for locally advanced UTUC significantly prolonged oncological prognosis. Multivariate Cox regression analysis using an IPTW model revealed that the impact of NAC was significant on CSS (HR, 0.37; 95%CI, 0.15–0.92; *P* = 0.031) but not on OS (HR, 0.46; 95%CI, 0.21–1.02; *P* = 0.056) [14]. Based on these results, the clinical impact of NAC on OS might be limited. Firstly, we thought that the rate of other cause of death might be higher in the NAC group. However, the number of other cause of deaths were not different between the Ctrl (*n* = 7, 5.3%) and the NAC (*n* = 5, 5.0%) groups. The next possible reason for this discrepancy might be the long-term study periods under the paradigm shift. We started NAC for locally advanced UTUC in selected patients after 2006 based on studies of bladder cancer indicating improved survival after NAC. After 2010, we expanded our inclusion criteria for all patients with locally advanced UTUC regardless of age and renal function. Therefore, short follow-up periods in the NAC group may have influence on the results. Therefore, further studies with long-term follow-up are needed on this issue.

The use of carboplatin in a neoadjuvant setting and the optimal number of courses still are being debated [15–20]. Because no evidence clearly supports the superiority of a cisplatin-based against a carboplatin-based regimen in a neoadjuvant setting in patients with UTUC [16, 21], we designed a strategy including carboplatin-based NAC followed by immediate surgery in patients with UTUC and impaired renal function. Because multiple studies have suggested that a delay of > 90 days in undergoing radical cystectomy is associated with adverse outcomes [22], we basically planned two courses of NAC and surgery within 90 days in accordance with the presence of muscle-invasive bladder cancer. Our study showed that there were no clear differences in tumor responses and prognosis between the two regimens. Although it is difficult to draw a definitive conclusion on the efficacy of a carboplatin-based regimen and optimal number of NAC courses, the potential activity of a carboplatin-based regimen is worth noting as it could be a viable option in patients with locally advanced UTUC who are unfit for cisplatin.

Although the body of evidence suggested a survival benefit of those based on the outcomes from muscle-invasive bladder cancer, to our knowledge no robust evidence exists to recommend the role of NAC for UTUC. Currently, only a few prospective randomized studies are ongoing for locally advanced UTUC. One ongoing randomized trial (NCT02876861) evaluating the role of 2 to 4 cycles of NAC (gemcitabine and cisplatin) for patients with locally advanced UTUC who are eligible for cisplatin hopefully will provide insight for clinical benefit. However, this study will not provide the useful information for the role of NAC in cisplatin-ineligible patients. The use of carboplatin in a neoadjuvant setting is still a matter of debate [14]. The present study showed that there were no clear differences in tumor responses and prognosis between the two regimens. Although the limitations of the present study prevent us from drawing definitive conclusions on the efficacy of a carboplatin-based regimen, it is worth noting the potential activity of carboplatin-based regimens as a viable option in patients with locally advanced UTUC who are unfit for cisplatin. On the other hand, efficacy and feasibility of immune-oncology therapy in cisplatin-ineligible patients have been reported and are promising [23]. The single-arm phase II IMvigor210 trial included 119 cisplatin-ineligible, treatment-naive patients with metastatic bladder cancer. The results suggested that the objective response rate with atezolizumab was 23.5% (*n* = 28; 95% CI, 16.2–32.2), including a complete response rate of 9% without severe toxicity. Although the clinical benefit of immune-oncology therapy in a neoadjuvant setting for treatment of UTUC remains unclear, these results encouraged us to see continued progress in the treatment of advanced urothelial carcinoma.

Response to salvage chemotherapy after recurrence may become concern about negative effect of NAC use. It is believed that the recurrent disease might be resistant to salvage chemotherapy when patients already received systemic (neoadjuvant) chemotherapy. However, recent study suggested that adjuvant chemotherapy after NAC and radical cystectomy may prolong OS among patients with locally advanced muscle invasive bladder cancer [24]. They investigated 788 patients with pT3-4 and/or pN+ bladder urothelial carcinoma and compared OS among patients who received NAC and radical cystectomy followed by adjuvant chemotherapy vs observation using IPTW-adjusted analyses. IPTW-adjusted Kaplan-Meier curves showed that median OS was significantly longer for NAC and radical cystectomy followed by adjuvant chemotherapy (29.9 months, 5-year OS rate: 36.8%) vs NAC and radical cystectomy followed by observation (24.2 months, 5-year OS rate: 24.7%) (*P* = 0.046). In the IPTW-adjusted Cox proportional hazards regression analysis, NAC and radical cystectomy followed by adjuvant chemotherapy was associated with a significant OS benefit (HR 0.78; *P* = 0.046). Although this study was retrospective, the patients with NAC shows better response to salvage chemotherapy after recurrence than those without. Therefore, response to secondary chemotherapy may be not worse in these patients with NAC.

Several limitations must be acknowledged, including the limited sample size and retrospective study design. We were unable to control selection bias and other unmeasurable confounders despite the use of statistical methods. We could not obtain the safety profiles in the patients receiving NAC. This long-term retrospective study included uncontrollable inherent factors such as the NAC use and/or laparoscopic surgery. Tumor downstaging was not evaluated by ureteroscopy. Due to the retrospective nature, the information for dissected lymph nodes was limited. Because no strong recommendation exists for template lymph node dissection, most patients underwent sampling dissection of regional lymph nodes alone. In addition, cN+ patients were not indicated or marginal for surgery approximately in a decade ago. Therefore, there were strong limitations in the exact information such as number of dissected lymph nodes and areas in the present study. Furthermore, it is difficult to draw a definitive conclusion of clinical benefit of carboplatin-based NAC for locally advanced UTUC because of the limited number of patients with selection bias. Regardless of these limitations, our study supported the potential benefit of NAC for locally advanced UTUC.

In conclusion, the platinum-based NAC for locally advanced UTUC potentially improves oncological outcomes. A carboplatin-based regimen might be a useful alternative in patients with UTUC who are ineligible for cisplatin. Further prospective randomized studies are needed to confirm the benefits of NAC in patients with locally advanced UTUC.

## MATERIALS AND METHODS

### Design and ethics statement

This retrospective, multicenter study was performed in accordance with the ethical standards of the Declaration of Helsinki and approved by an ethics review board of Hirosaki University School of Medicine (authorization numbers; 2017–067) including all other hospitals. All hospitals approved the present study.

### Patient selection

Between February 1995 and April 2017, we performed radical nephroureterectomy (RNU) in 426 consecutive patients with UTUC at the Hirosaki University Hospital, Aomori Rosai Hospital, Mutsu General Hospital, Tsugaru General Hospital, and Aomori Prefectural Central Hospital. The indications for NAC were locally advanced high-risk UTUC, including stages cT3–4 or locally invasive cN+ disease. We identified 234 high-risk patients who received 2 to 4 courses of NAC (NAC group) or surgery alone (Ctrl group).

### Evaluation of variables

The variables analyzed were age, sex, Eastern Cooperative Oncology Group performance status (ECOG PS), history of cardiovascular disease (CVD), hypertension, diabetes mellitus (DM), smoking, presence of hydronephrosis, regimen of chemotherapy, clinical stage, primary tumor site, laparoscopic surgery, tumor recurrence (intravesical superficial tumor and/or visceral metastasis), and renal function. Renal function was evaluated using eGFR by a modified version of the abbreviated Modification of Diet in Renal Disease Study formula for Japanese patients [25]. Toxicity was recorded prospectively using the National Cancer Institute Common Terminology Criteria for Adverse Events version 3.0. Tumor response was analyzed using Response Evaluation Criteria in Solid Tumors version 1.1. RFS, CSS, and OS were defined from the day of first treatment to the date of event onset.

### Neoadjuvant chemotherapy (NAC)

A regimen was selected based on the guidelines regarding eligibility for the proper use of cisplatin [26]. All urothelial cancer patients underwent chemotherapy at hospitalization. Most patients received either gemcitabine 800 to 1000 mg/m^2^ on days 1, 8, and 15 plus cisplatin 70 mg/m^2^ (GCis) on day 2 every 3 weeks, or gemcitabine 800 to 1000 mg/m^2^ on days 1, 8, and 15 plus carboplatin (GCarbo) at an area under the curve of 4 to 4.5 according to the Calvert formula on day 2 every 3 weeks, for 2–4 cycles [19, 27]. Only a few patients underwent standard dose of MVAC (methotrexate: 30 mg/m^2^, days 1, 15, 22; vinblastine: 3 mg/m^2^, days 2, 15, 22; doxorubicin: 30 mg/m^2^, day 2; and cisplatin: 70 mg/m^2^, day 2) or docetaxel-based regimen (docetaxel: 75 mg/m^2^, day 1; ifosfamide: 2.0 g/m^2^, days 1–3 and nedaplatin: 75 mg/m^2^, day 2).

Tumor response was evaluated during the second course of NAC. To reduce the delay of surgery, we basically planned two courses of NAC and surgery within 90 days in accordance with the presence of muscle-invasive bladder cancer [22]. Patients with insufficient tumor response (stable or progressive disease) received 3 or 4 cycles of NAC.

### Surgical procedure

Open or laparoscopic nephroureterectomy, which included removal of the kidney, ureter, and ipsilateral bladder cuff, was performed [28]. The distal ureter was managed via the extravesical approach. A sampling dissection of regional lymph nodes was performed depending on the tumor stage. Postoperative complications were reviewed using the Clavien–Dindo classification.

### Patient follow-up

After treatment, each patient was assessed every 3 to 6 months using a blood and serum test, ultrasonography, urine cytology, cystoscope, and computed tomography (CT) for the detection of tumor recurrence. Adjuvant chemotherapy was not administered routinely. Salvage therapy was indicated when recurrent disease was detected by CT.

### Outcome evaluations

We retrospectively evaluated the tumor response, post-therapy pathological downstaging (cT–pT stage), and LVI between the Ctrl and NAC groups. Intravesical RFS, visceral RFS, CSS, and OS were evaluated using the Kaplan–Meier methods with a log-rank test between the two groups. Multivariate Cox regression analysis was performed for independent factors for RFS, CSS and OS. Additionally, we analyzed the impact of regimen for the NAC group on oncological outcomes between the GCis and GCarbo groups.

### Statistical analysis

The clinical data were analyzed statistically using SPSS version 24.0 (SPSS, Inc., Chicago, IL, USA), GraphPad Prism 5.03 (GraphPad Software, San Diego, CA, USA), and R 3.3.2 (The R Foundation for Statistical Computing, Vienna, Austria). Categorical variables were compared using Fisher's exact test or the χ^2^ test. Quantitative variables were expressed as mean with standard deviation or median with IQR. The difference between the groups was compared statistically using Student's *t*-test for a normal distribution or the Mann–Whitney *U* test for a non-normal distribution. *P* values < 0.05 were considered statistically significant. We used multivariate analyses with the Cox regression model and HR with 95% confidence interval (CIs) were calculated. We also performed IPTW Cox regression analysis for prognosis in the high-risk (stages cT3-4 or cN+ disease, *n* = 234) patients. The IPTW method reweights exposed and unexposed groups to emulate a propensity score-matched population [29]. Variables included in the IPTW model were age, sex, eGFR, cT, and cN.

### Ethical standards

This study was performed in accordance with the ethical standards of the Declaration of Helsinki and approved by an ethics review board of Hirosaki University School of Medicine (authorization numbers; 2017-067) and all other hospitals.
